# Connected speech features in non-English speakers with Alzheimer’s disease: protocol for scoping review

**DOI:** 10.1186/s13643-023-02379-y

**Published:** 2024-01-25

**Authors:** Arpita Bose, Samrah Ahmed, Yesi Cheng, Aida Suárez-Gonzalez

**Affiliations:** 1 School of Psychology and Clinical Language Sciences, University of Reading, Reading, UK; 2 Nuffield Department of Clinical Neurosciences, University of Oxford, Oxford, UK; 3 Wexham Park Hospital, NHS Frimley Health Foundation Trust, Slough, UK; 4 Dementia Research Centre, UCL Queen Square Institute of Neurology, University College London, London, UK

**Keywords:** Alzheimer’s disease, Dementia, Connected speech analysis, Spontaneous speech, Naturalistic speech, Lexicon, Grammar, Syntax, Narrative

## Abstract

**Background:**

A large body of literature indicates that connected speech profiles in patients with Alzheimer’s disease (AD) can be utilized for diagnosis, disease monitoring, and for developing communication strategies for patients. Most connected speech research has been conducted in English, with little work in some European languages. Therefore, significant drawback remains with respect to the diversity of languages studied, and how the fragmentation of linguistic features differs across languages in AD. Accordingly, existing reviews on connected speech in AD have focused on findings from English-speaking patients; *none* have specifically focused on the linguistic diversity of AD populations. This scoping review is undertaken to provide the currently reported characteristics of connected speech in AD in languages other than English. It also seeks to identify the type of assessments, methods to elicit speech samples, type of analysis and linguistic frameworks used, and micro- and macro-linguistic features of speech reported in non-English speakers with AD.

**Method:**

We will conduct a scoping review of published studies that have quantitively assessed connected speech in AD in languages other than English. The inclusion criteria for the studies would be subject/s with a clinical diagnosis of AD. The search will include the electronic databases PubMed, Ovid-Embase, PsycINFO, Linguistic and Language Behaviour Abstracts (LLBA), and Web of Science up until March 2023. Findings will be mapped and described according to the languages studied, the methodology employed (e.g., patient characteristics, tasks used, linguistic analysis framework utilized), and connected speech profiles derived (e.g., micro- and macro-linguistic reported).

**Discussion:**

The scoping review will provide an overview of languages studied in connected speech research in AD with variation in linguistic features across languages, thus allowing comparison with the established key features that distinguish AD patients from healthy controls. The findings will inform future research in connected speech in different languages to facilitate robust connected speech research in linguistically and ethnically diverse populations.

**Supplementary Information:**

The online version contains supplementary material available at 10.1186/s13643-023-02379-y.

## Background and introduction

From a clinical point of view, typical AD is characterized by insidious and progressive onset and a cognitive profile where the initial and most prominent deficit is impairment in episodic memory, including learning and recall of recently learned information [14]. However, a burgeoning body of research has shown that changes in connected speech (i.e., spoken language used in continuous sequence) are among the earliest signs of cognitive decline in AD [1, 11, 16] and that these changes progress through the successive clinical stages of the disease (e.g., [1, 10, 15, 16]. This knowledge reveals the potential value of connected speech analysis in the early identification and monitoring of AD. Moreover, unpacking the linguistic and cognitive mechanisms underlying the breakdown of connected speech in AD is critical for the development of new communication therapies to assist patients, families, and health and care professionals during the dementia journey. Indeed, support to overcome communication difficulties is among the core recommendation included in the NICE Dementia Care Pathway [18]. Supporting communication is one of the core principles underlying “Living Well” and “Supporting Well,” key pillars of the NHS England Dementia Wellbeing Pathway and the UK National Dementia strategy [8].

Successful production of connected speech involves simultaneous use and coordination of several linguistic (e.g., morpho-syntactic, lexico-semantic, phonetic, phonological, pragmatic) and cognitive processes (e.g., memory, attention, executive function, speed of processing). Therefore, it provides an opportunity to identify specific levels of linguistic deficits from spoken output. Recent literature reviews on characteristics of connected speech in AD point to a pattern of deficit in several linguistic levels including speech rate, syntactic structure and complexity, lexical content, semantic content and efficiency, as well as spontaneity and fluency of speech [6, 9, 15, 25]. Specifically, the key features that distinguish AD from healthy control participants are as follows: reduced speech rate and spontaneity including increased repetitions and revisions,simplified syntax and sentence structures including shorter and grammatically simpler sentences; word finding difficulties and increased use of pronouns (i.e., over production of *he, she, they,* and *it*, rather than the use of specific nouns); inflectional errors in nouns and verbs (e.g., difficulty producing verb tense-*play, plays, played, playing*); and reduced semantic content and uninformative speech with low idea density and efficiency. The progress in the field has been encouraging; however, a significant drawback remains with regard to the diversity of languages studied, and how fragmentation of linguistic features differs across different languages [5].

Our understanding of linguistic breakdown in AD is limited as most studies have been conducted in English-speaking participants and handful of other European languages [6, 9, 23]. This is far from capturing the structural and typological diversity of languages spoken in the world. Cross-linguistic research in language impairments has shown that impairments are determined by the structure of the language system [19]. Research indicates that features of language impairment and specific diagnostic markers in AD depend on the structure of the language itself [3, 7, 12]. That is, there are distinct differences in how language impairment in AD manifests itself in English compared to other languages. To illustrate, our own research has shown that Bengali-speaking AD patients produce fewer pronouns, in direct contrast with the overuse of pronouns by English-speaking AD patients [7]. Furthermore, these patients did not show difficulty producing verb tenses, which is among the common diagnostic features in English-speaking patients [1]. Similarly, Kavé and Levy [12] reported that Hebrew-speaking AD patients produced a similar proportion of inflected words compared to controls in Cookie Theft picture description, a difference that is typically found in English-speaking AD patients [1, 24]. These are not idiosyncratic findings. Rather, it highlights that the features that are impaired in AD depend on the nature of the language itself.

Several reviews on connected speech in AD have focused on findings from English-speaking patients, and *none* have specifically focused on linguistic diversity of AD populations. We will therefore conduct a scoping review [17] to map the literature regarding connected speech in AD across non-English languages. The specific purposes of this scoping review are as follows: (1) to identify the breadth and extent of connected speech literature in non-English speakers with AD, (2) to determine their methodological characteristics; (3) to identify impaired linguistic features currently described across different linguistic levels, and (4) to identify language-specific features.

## Methods/design

We will follow Preferred Reporting Items for Systematic Reviews and Meta-Analyses-Extension for Scoping Reviews (PRISMA-ScR) guidelines for scoping review publication [26]. The literature will be systematically scoped following the framework outlined by Peters et al. [22], based on the framework proposed by Arksey and O’Malley [2]. The five essential steps include the following: (1) Identifying the research questions, (2) Identifying relevant studies, (3) Study selection, (4) Charting the data, (5) Collating, summarizing, and reporting the results (see Additional file 1: PRISMA-ScR checklist).

### Identifying the research questions

This scoping review aims to address the following objectives and specific research questions:

Objective 1: Breadth and extent of connected speech literature in non-English speakers with AD.

1. How many studies have evaluated differences in connected speech characteristics in individuals with AD and control speakers in languages other than English? Which languages have been studied?
2. What are the study characteristics in terms of sample size, dementia diagnosis, and severity criteria?

Objective 2: To determine their methodological characteristics.

1. What tasks are used to elicit connected speech?
2. Which linguistic framework and/or analysis tool being used to analyze the connected speech samples?

Objective 3: To identify impaired linguistic features in non-English speakers with AD.

1. What are the linguistic levels investigated?
2. Which micro- and macro-linguistic features are identified in these studies?
3. Do these findings map onto any language-specific characteristics?

Objective 4: To identify language-specific features.

1. What are the language-specific connected speech features reported in non-English languages?

### Identifying relevant studies

#### Information sources and search terms

The choice of databases to be included in this scoping review was guided by recommendation in the literature [4] and developed in consultation with an expert research librarian. The search will be conducted using PubMed, Ovid-Embase, PsycINFO, Linguistic and Language Behaviour Abstracts (LLBA), and Web of Science with an unlimited starting date, up until October 2023. The literature included will be indexed, published, and peer-reviewed articles written in the English language. Database-specific conventions and the use of multiple search fields and filters will be customized for individual databases. In addition to the database searches, reference lists will be checked from key articles and reviews.

The search strategy will involve the steps recommended by Peters et al. [20]. Search terms were developed by identification of key words from relevant articles, pilot searches, and in consultation with experienced researchers and librarians. Following search terms will be used for PubMed: (“natural language”[TW] OR “natural discourse”[TW] OR “Speech” (MeSH Terms) OR “oral communication”[TW] OR “speech”[TW]) AND (“Alzheimer disease”[MeSH Terms] OR Alzheimer*[TW]). See Additional file 2 for an example of detailed search strategy for PubMed.

#### Data management and screening

EndNote20 version will be used to export and manage the results from the search. After merging search results from different databases, duplicates will be identified and removed in Endnote using the default settings in “Find duplicates.” The screening of the candidate articles and the selection of qualified studies will be conducted using a multi-level title-first method [13]. First, the primary reviewer (YC) will independently inspect all the citations, while reviewers (AB and SA) will independently inspect 50% of the citations each, so that every item is considered by at least two independent reviewers. Screening will take place first by title, and then by abstract. Any differences in the agreement will discussed and resolved by consultation with AB and ASG. The selected full text versions will be then screened to ascertain whether they meet the pre-decided eligibility criteria for inclusion (see next section, Study Selection, for a list of inclusion and exclusion criteria) to produce the final list of articles to be included in the review. At full text screening, a 20% sample of the articles selected by the primary reviewer will be screened by reviewers (AB, ASG) to ensure the reliability of selection. Where there is a conflict of interest, for example where a member of the review team is an author on a considered article, that team member will be excluded from the decision-making process for the articles in question.

### Study selection 

#### Population

Subjects with a clinical diagnosis of AD (i.e., the studies are required to specify the clinical criteria used for clinical diagnosis) will be included. If studies are reporting on mixed populations, they will be included if at least one participant is diagnosed with AD, and if this data was separately identified. There will be no limitations on age, gender, severity of dementia, or ethnicity.

#### Concepts

Studies that investigated connected speech in AD patients to determine profile micro-linguistic and/or macro-linguistic features will be included.

#### Context

Study selection will focus on articles published in English and where full text is available. Any articles that do not present new findings or original research, such as review papers or editorials, will not be included, and neither will conference abstracts and opinion pieces be included. Articles could include quantitative, qualitative, mixed method, or case research studies. In some studies, comparison may be made between groups. We included studies irrespective if the participants reported in the research are monolingual, bilingual, or multilingual. Multiple reports that use data from the same study will be identified and collated to avoid duplication of findings.

#### Exclusion criteria

1. Studies not including participants with AD.
2. Studies that did not measure/assess at least one micro-linguistic or macro-linguistic features in connected speech.
3. Studies that tested only English-speaking AD patients.
4. Articles where full text is not readily available or is not in English.
5. Studies where no original findings are presented (e.g., reviews, editorials, opinion pieces, letters to the editor).
6. Non-peer-reviewed material.
7. Conference abstracts.
8. Errata/correction of no significance to required data.
9. Exclusion decisions and characteristics of excluded studies will be recorded. The search findings will be presented in a PRISMA flow diagram [26].

### Charting the data: data extraction and management

Peters et al. [21] best practise guidance for reporting items for scoping review would be followed. Information extracted from each article will be recorded in a tailored data-charting form on Microsoft Excel including the following:

1. Metadata: Authors and publication date
2. Study population and design
  ◦ Study populations (clinical diagnosis of AD and controls)
  ◦ Severity of dementia
  ◦ Sample size
  ◦ Age, gender, educational attainment
  ◦ Language status (monolingual or bilingual)
  ◦ Study design
  ◦ Setting
3. Connected speech features
  ◦ Language of testing
  ◦ Protocol and task/s used to elicit connected speech
  ◦ Transcription (manual and/or automatic)
  ◦ Data analysis (manual and/or automatic)
  ◦ Type of analysis and linguistic framework used
  ◦ Target linguistic levels analyzed
  ◦ Specific micro-linguistic features and variables reported (e.g., syntactic complexity, sentence length, proportion of nouns or pronouns, inflectional indices)
  ◦ Specific macro-linguistic features and variables reported (e.g., coherence, correct information units)
  ◦ Key findings: A “key finding” will correspond to at least one statistically significant association or difference across groups for at least one outcome measures of the relevant category [9].

In keeping with published scoping review guidance, we will not formally appraise the quality of the included studies.

### Collating, summarizing and reporting the results

Overall results will be tabulated, and numerical and descriptive summary will be developed based to address the research questions. A summary will be developed focusing on the connected speech characteristics of non-English speaking AD with emphasis on identifying language-specific characteristics of connected speech. The data will be explored, with a focus on describing the linguistic levels examined in non-English AD patients and the linguistic impairments reported across domains. A modified version of the charting form from the JBI template [22] will be used to extract data from each study (see Additional file 3: Charting form). Experts in AD research, speech and language therapist working in memory clinics as well as people with dementia and their families will be consulted to inform directions for future research.

### Dissemination and ethics

The completed review will be submitted for journal publication. Preliminary findings will be presented at relevant conferences. Ethical approval is not required for this study.

## Discussion

The scoping review will provide an overview of languages studied in connected speech research in AD along with variation in linguistic features across languages. This will allow comparison with the established key features of English-connected speech that distinguish AD patients from healthy controls. In doing so, there will be an opportunity to identify gaps and limitations in current research in connected speech in AD, and also understanding the nature of the research to date and summarize its findings. The outcomes from this review can be of use to inform the design of connected speech research in different languages and facilitate robust connected speech research in linguistically and ethnically diverse populations.

### Supplementary Information

**Additional file 1.** PRISMA-ScR checklist.

**Additional file 2.** Search strategy.

**Additional file 3.** Charting form.

## Data Availability

Data sharing is not applicable to this article as no datasets will be generated or analyzed during the current study.

## Abbreviation

AD: Alzheimer’s disease
