# Supplementary material for: Connected speech features in non-English speakers with Alzheimer’s disease: protocol for scoping review
Source: Syst Rev. 2024 Jan 25;13:40. doi: 10.1186/s13643-023-02379-y (PMC10809489; doi:10.1186/s13643-023-02379-y)
Supplement: Supplementary file 2 — Additional file 2. Search strategy. [file 13643_2023_2379_MOESM2_ESM.docx]

**Additional File 2**

**Search strategy**

The specific purposes of this scoping review are: 1) to identify the breath and extent of *connected speech* literature in *non-English* speakers with *AD*; 2) to determine their methodological characteristics; 3) to identify impaired linguistic features currently described across different linguistic levels, and 4) to identify language-specific features.

The proposed search strategy has been developed in consultation with university librarian to ensure key articles are captured. The initial search will involve 5 databases: PubMed, Ovid-Embase, PsycINFO, Linguistic and Language Behaviour Abstracts (LLBA), and Web of Science. The resulting reference lists of identified articles will then be searched for additional studies.

An example of the search to be carried out in PubMed is shown below.

**PubMed search**

The search strategy involves entering search terms using the PubMed default [All Fields] setting within the Advanced Search function, having cleared all default filters. Using the [All Fields] setting in PubMed draws upon Medical Subject headings (MeSH) without limiting to specific qualifiers or subheadings.

**Concept 1:**

(“natural language”[TW] OR “natural discourse”[TW] OR “Speech” (MeSH Terms) OR “oral communication”[TW] OR “speech”[TW])

**Concept 2:**

(“Alzheimer disease”[MeSH Terms] OR Alzheimer*[TW])

1. (“natural language”[TW] OR “natural discourse”[TW] OR “Speech” (MeSH Terms) OR “oral communication”[TW] OR “speech”[TW])
2. (“Alzheimer disease”[MeSH Terms] OR Alzheimer*[TW])
3. 1 AND 2

**PubMed search string**

("natural language"[TW] OR "discourse"[TW] OR "Speech"[MeSH Terms] OR "oral communication"[TW] OR "speech"[TW]) **AND**("Alzheimer disease"[MeSH Terms] OR Alzheimer*[TW])

Resulting search details for PubMed (sorted by default **Best Match**):

#1 Search: **"natural language"[TW] OR "natural discourse"[TW] OR "Speech" (MeSH Terms) OR "oral communication"[TW] OR "speech"[TW]**

(("natural language"[Text Word] OR "natural discourse"[Text Word] OR "Speech"[All Fields]) AND (("medical subject headings"[MeSH Terms] OR ("medical"[All Fields] AND "subject"[All Fields] AND "headings"[All Fields]) OR "medical subject headings"[All Fields] OR "mesh"[All Fields]) AND "Terms"[All Fields])) OR "oral communication"[Text Word] OR "Speech"[Text Word]

**Translations**

**MeSH:** "medical subject headings"[MeSH Terms] OR ("medical"[All Fields] AND "subject"[All Fields] AND "headings"[All Fields]) OR "medical subject headings"[All Fields] OR "mesh"[All Fields]

#2 Search: **("Alzheimer disease"[MeSH Terms] OR Alzheimer*[TW])**

"Alzheimer disease"[MeSH Terms] OR "alzheimer*"[Text Word]
