# Supplementary material for: Connected speech features in non-English speakers with Alzheimer’s disease: protocol for scoping review
Source: Syst Rev. 2024 Jan 25;13:40. doi: 10.1186/s13643-023-02379-y (PMC10809489; doi:10.1186/s13643-023-02379-y)
Supplement: Supplementary file 3 — Additional file 3. Charting form. [file 13643_2023_2379_MOESM3_ESM.docx]

**Additional file 3: Charting form**

| **Scoping Review Details** | | |
| --- | --- | --- |
| Scoping review title | Connected speech features in non-English speakers with Alzheimer’s Disease: protocol for scoping review | |
| **Objectives** | 1. To identify breadth and extent of connected speech literature in non-English speakers with AD. 2. To determine their methodological characteristics 3. To identify impaired linguistic features in non-English speakers with AD 4. To identify language-specific features | |
| **Research questions** | *Objective 1*   1. How many studies have evaluated differences in connected speech characteristics in individuals with AD and control speakers in languages other than English? Which languages have been studied? 2. What are the study characteristics in terms of sample size, dementia diagnosis and severity criteria?   *Objective 2*   1. What tasks are used to elicit connected speech? 2. Which linguistic framework and/or analysis tool being used to analyse the connected speech samples?   *Objective 3*   1. What are the linguistic levels investigated? 2. Which micro- and macro-linguistic features are identified in these studies? 3. Do these findings map onto any language-specific characteristics?   *Objective 4*   1. What are the language-specific connected speech features reported in non-English languages? | |
| **Evidence source Details and Characteristics** | | |
| Authors, year of publication | |  |
| Title, Journal | |  |
| Study design (e.g. case-control; cross-sectional) | |  |
| Participants (details e.g. sample size, dementia types, languages spoken etc) | |  |
| Setting | |  |
| **Details/Results extracted from source of evidence**(in relation to the concept of the scoping review) | | |
| Language of testing | |  |
| Severity of language impairment(s) | |  |
| Protocol and task/s used to elicit connected speech | |  |
| Transcription (manual and/or automatic) | |  |
| Data analysis (manual and/or automatic) | |  |
| Type of analysis and linguistic framework used | |  |
| Target linguistic levels analyzed | |  |
| Specific micro-linguistic features and variables reported (e.g., syntactic complexity, sentence length, proportion of nouns or pronouns, inflectional indices) | |  |
| Specific macro-linguistic features and variables reported (e.g., coherence, correct information units) | |  |
| Key findings | |  |
| Significant difference the variables between AD and control participants | |  |
